# Supplementary material for: A Triphenylphosphonium-Functionalized Delivery System for an ATM Kinase Inhibitor That Ameliorates Doxorubicin Resistance in Breast Carcinoma Mammospheres
Source: Cancers (Basel). 2023 Feb 25;15(5):1474. doi: 10.3390/cancers15051474 (PMC10000448; doi:10.3390/cancers15051474)
Supplement: Supplementary file 1 [file cancers-15-01474-s001.zip › cancers-2080166-supplementary.pdf]

Supplementary Material

A triphenylphosphonium functionalized delivery system of an ATM kinase inhibitor ameliorates doxorubicin resistance in breast carcinoma mammospheres

Venturina Stagni[1,2,*], Archontia Kaminari[3], Claudia Conadini[2,], Daniela Barilà[2,4], Rosario Luigi Sessa[1,2], Zili Sideratou[3], Spiros A. Vlahopoulos[5], Dimitris Tsiourvas[3]

[1] Institute of Molecular Biology and Pathology, National Research Council (CNR), Rome, Italy
[2] Laboratory of Cell Signaling, IRCCS, Fondazione Santa Lucia, Rome, Italy
[3] Institute of Nanoscience and Nanotechnology, NCSR ''Demokritos'', Aghia Paraskevi, Greece
[4] Department of Biology, University of Tor Vergata, Rome, Italy
[5] Horemeio Research Laboratory, First Department of Paediatrics, N.K.U.A, Athens, Greece
* Correspondence: venturina.stagni@cnr.it

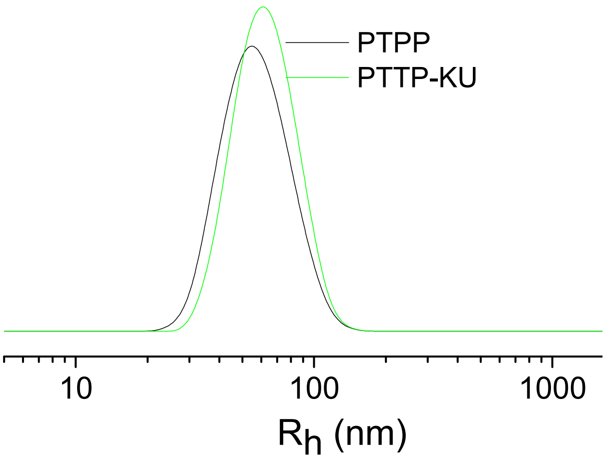

Figure S1. Intensity weighted hydrodynamic size distribution of PTPP and PTPP-KU nanoparticles in RPMI-1640 media supplemented with 10% Fetal Bovine Serum.

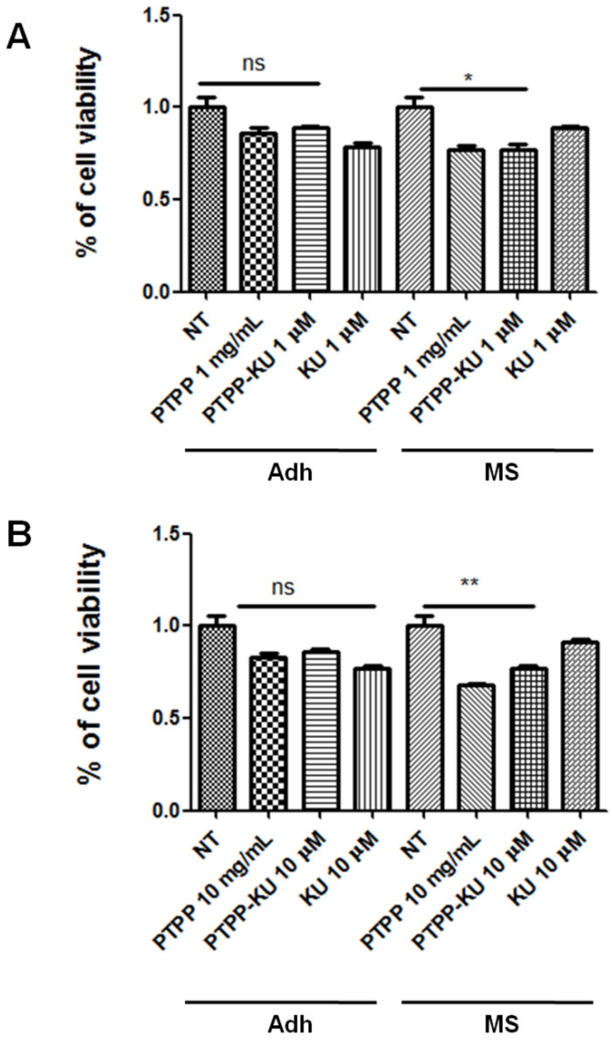

Figure S2. Cell viability of the MCF-7 cell line grown in adherent (Adh) and in mammospheres (MS) conditions. Cells were treated with low (A) or high (B) doses of free KU-55933 (KU), PTPP-KU and PTPP as indicated. After 24 hours cell viability was measured with MTS assay. Results are expressed as the mean±s.d. for at least three independent experiments and analyzed using Student's t-test (* $p < 0.05$, ** $p < 0.01$, ns not significant).

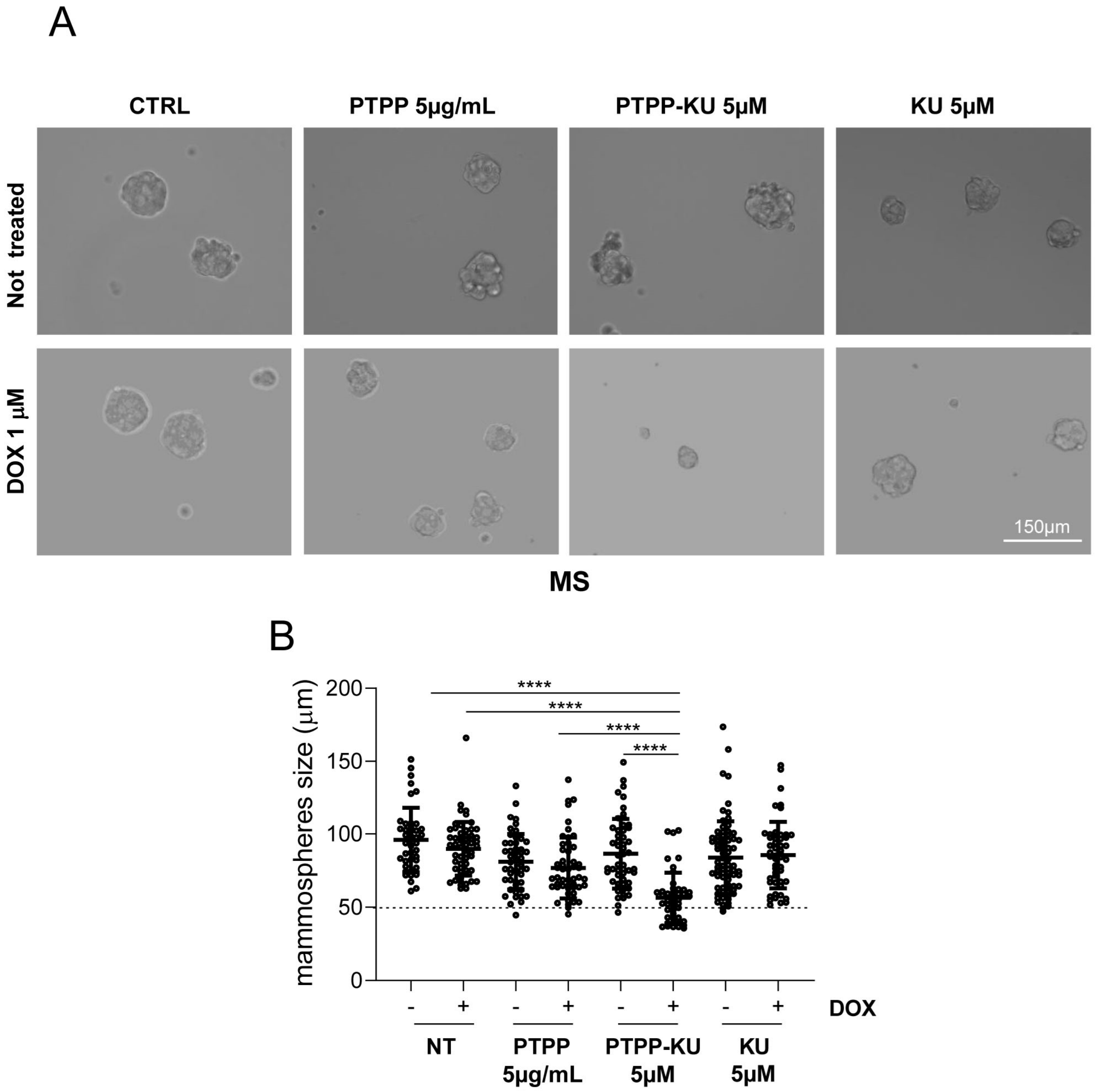

Figure S3. (A) Optical microscopy images (20X) representative of mammospheres' morphology after treatment with 1 µM of DOX for 3 hours and free KU (5 µM), PTPP-KU (5 µM) and PTPP (5 µg/mL), administered 30 min before adding DOX; scale bar 150 µm. (B) Graph representing the diameters of mammospheres. The results are expressed as the mean ± SD for at least three independent experiments and were analyzed using a Student's *t*-test (**** $p < 0.0001$; statistical analysis is not shown if it is not considered significant) ($n = 4$).

NT	DOX	PTPP-KU 5uM	PTPP-KU 10uM	PTPP 5ug/uL	PTPP 10ug/uL	KU 5uM	KU 10uM
400	450	600	1200	500	600	430	500
398	456	700	1300	520	700	450	600
429	500	680	1200	555	650	460	500

NT	DOX	PTPP-KU 5uM	PTPP-KU 10uM	PTPP 5ug/uL	PTPP 10ug/uL	KU 5uM	KU 10uM
97,44841	151,2314	183,1285	260,9008	242,343	298,2827	148,3825	138,1584
105,3827	162,201	172,079	193,5389	160,923	372,6736	141,1138	159,1924
112,6248	127,7213	209,3543	239,5474	164,118	281,4289	183,2083	159,6184
107,1933	172,3186	147,2909	261,3801	171,0406	326,1593	126,6829	136,907
96,1704	103,4125	178,9483	228,2583	184,1402	272,7491	132,6736	160,0444
94,70601	176,0195	145,4537	217,2887	180,7056	364,3399	140,2618	152,7757
108,6576							
87,03794							
107,6991							
97,18216							
98,40692							
87,49057							

Figure S4. Raw data of Figure 3.

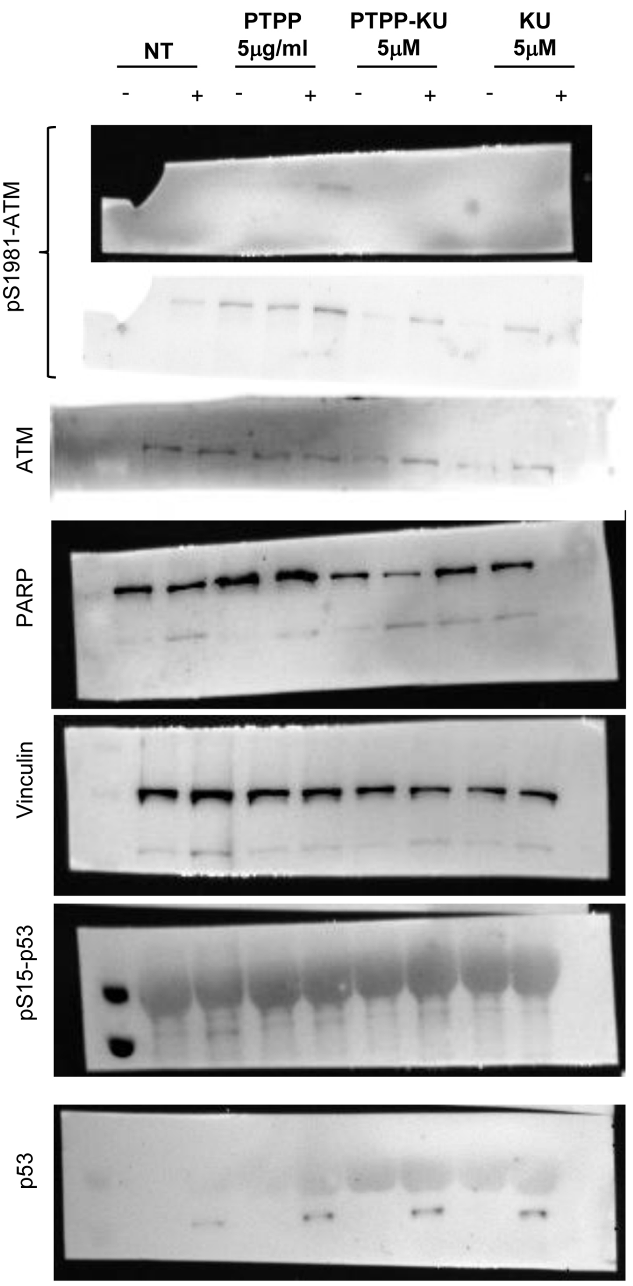

Figure S5. Original Western Blot.
